# p16^INK4a^ and its regulator miR-24 link senescence and chondrocyte terminal differentiation-associated matrix remodeling in osteoarthritis

**DOI:** 10.1186/ar4494

**Published:** 2014-02-27

**Authors:** Didier Philipot, David Guérit, Daniela Platano, Paul Chuchana, Eleonora Olivotto, Francisco Espinoza, Anne Dorandeu, Yves-Marie Pers, Jacques Piette, Rosa Maria Borzi, Christian Jorgensen, Danièle Noel, Jean-Marc Brondello

**Affiliations:** 1 INSERM U844, CHU St Eloi, Bat INM, 80 av A. Fliche, 34298 Montpellier, cedex 05, France; 2 Université de Montpellier-1, UFR de Medecine, F-34000, Montpellier, France; 3 Laboratorio di immunoreumatologia e rigenerazione tissutale, IOR, Istituto di ricerca Codivilla Putti, I-40136 Bologna, Italy; 4 Service de Medecine légale CHU La Peyronie, F34000 Montpellier, France; 5 Unité clinique, thérapeutiques des maladies des os et articulations, CHU Lapeyronie, F34000 Montpellier, France; 6 UMR5237, CNRS, CRBM, 1919 route de Mende, F34295 Montpellier, France

## Abstract

**Introduction:**

Recent evidence suggests that tissue accumulation of senescent p16^INK4a^-positive cells during the life span would be deleterious for tissue functions and could be the consequence of inherent age-associated disorders. Osteoarthritis (OA) is characterized by the accumulation of chondrocytes expressing p16^INK4a^ and markers of the senescence-associated secretory phenotype (SASP), including the matrix remodeling metalloproteases MMP1/MMP13 and pro-inflammatory cytokines interleukin-8 (IL-8) and IL-6. Here, we evaluated the role of p16^INK4a^ in the OA-induced SASP and its regulation by microRNAs (miRs).

**Methods:**

We used IL-1-beta-treated primary OA chondrocytes cultured in three-dimensional setting or mesenchymal stem cells differentiated into chondrocyte to follow p16^INK4a^ expression. By transient transfection experiments and the use of knockout mice, we validate p16^INK4a^ function in chondrocytes and its regulation by one miR identified by means of a genome-wide miR-array analysis.

**Results:**

p16^INK4a^ is induced upon IL-1-beta treatment and also during *in vitro* chondrogenesis. In the mouse model, *Ink4a* locus favors *in vivo* the proportion of terminally differentiated chondrocytes. When overexpressed in chondrocytes, p16^INK4a^ is sufficient to induce the production of the two matrix remodeling enzymes, MMP1 and MMP13, thus linking senescence with OA pathogenesis and bone development. We identified miR-24 as a negative regulator of p16^INK4a^. Accordingly, p16^INK4a^ expression increased while miR-24 level was repressed upon IL-1-beta addition, in OA cartilage and during *in vitro* terminal chondrogenesis.

**Conclusions:**

We disclosed herein a new role of the senescence marker p16^INK4a^ and its regulation by miR-24 during OA and terminal chondrogenesis.

## Introduction

Tissue loss of function and integrity are inherent to aging and age-related disease onset. Because senescent p16^INK4a^-positive cells accumulate within numerous tissues throughout life [1], recent strong evidence suggested that these cells contribute to tissue degeneration by sustaining chronic inflammation and extracellular matrix remodeling [2]. Indeed, p16^INK4a^-positive cells exhibit a specific secretome called SASP (senescence-associated secretory phenotype) including pro-inflammatory cytokines (such as interleukin-6 (IL-6), IL-8, and IL-1β) and matrix remodeling regulatory metalloproteases (such as MMP1 and MMP13) [2]. Remarkably, specific conditional elimination of these cells in a premature aging murine model has revealed their essential role in the onset of several age-related diseases [3]. Interestingly, *Ink4a*, which encodes an archetypical cyclin-dependent inhibitor (CKI) associated with senescence, is also known to participate in terminal differentiation onset of several cellular lineages [4,5].

Osteoarthritis (OA) is a chronic degenerative disease characterized by progressive cartilage erosion and lesions in subchondral bone as well as in other joint tissues [6]. The anabolic function of chondrocytes, the major cellular component of articular cartilage, decreases with disease progression. This loss of function is associated mainly with an accumulation of p16^INK4a^-positive articular chondrocytes [7] harboring short telomeres [8] but also features of hypertrophic/terminally differentiated cells [9,10]. The latter is normally associated with endochondral ossification process during bone development [9,10]. Although OA regulatory mechanisms remain under investigation, it's now believed that articular mature chondrocytes in response to either inflammatory cytokines or aberrant developmental signals exemplified by Notch activation [10] are producing matrix remodeling enzymes (MMP1 and MMP13) and inflammatory cytokines (IL-8 and IL-6) [11,12]. All of these factors are deleterious for cartilage integrity. Therefore, OA is a multi-factorial complex disease in which articular chondrocytes exhibit characteristics of senescent-like and hypertrophic-like cells secreting SASP factors leading to impaired anabolic capacities [7]. Moreover, a reduction of p16^INK4a^ expression by RNA interference in OA chondrocytes was shown to lead to their functional rescue [13]. These results demonstrate a deleterious role for this senescence-associated CKI on articular chondrocytes. It remains to be understood how p16^INK4a^ increased expression occurs and could contribute to OA progression.

MicroRNAs (miRs) are small non-coding RNAs that are part of the miRNA-induced silencing complex (RISC) [14] and are involved in the regulation of gene expression. MiRs are key regulators of numerous physiological processes that are deregulated in pathological conditions [15], in particular OA [16,17]. Among miRs identified in OA, miR-22 targets BMP7, a factor inducing chondrocyte terminal differentiation [18]; miR-140 targets HDAC4, a histone deacetylase inducer of chondrocyte terminal differentiation [19,20]; and miR-27b targets MMP13, a key remodeling enzyme in hypertrophic terminally differentiated chondrocyte [21]. So far, none of these miRs has been found to be regulators of p16^INK4a^-associated senescent phenotypes during OA progression.

In this study, we demonstrate that p16^INK4a^ accumulates not only in response to inflammatory stimuli but also during chondrogenesis. *Ink4a* participates in cell cycle exit required for chondrocyte terminal differentiation onset during endochondral ossification. Moreover, p16^INK4a^ overexpression is sufficient to trigger MMP1 and MMP13 production in mature chondrocytes. By genome-wide microRNA array, we identify miR-24 as a regulator of p16^INK4a^ in chondrocytes. As expected, miR-24 is repressed in IL-1β-treated chondrocytes, in cartilages of patients with OA but also at the end of chondrogenesis while p16^INK4a^ accumulates. Finally, downregulation of miR-24 by an antagomir approach in primary chondrocytes leads to an increase in p16^INK4a^ expression and MMP1 secretion. Taken together, these data reveal for the first time that the senescent marker p16^INK4a^ and its epigenetic regulator miR-24 are reciprocally involved in both OA and bone developmental-associated matrix remodeling secretomes.

## Materials and methods

### Cell culture, chondrocytes, mesenchymal stem cells, cartilage samples, and mouse models

Primary human chondrocytes were isolated from cartilage of 11 OA patients (mean age of 62 years) undergoing knee arthroplasty after informed written consent from patients and approval by the local and national ethics committee (‘Cellule de bioéthique de la direction générale pour la recherche et innovation, Ministère de l’Enseignement Supérieur et de la Recherche’; registration number DC-2009-1052) were obtained, as described previously [22]. Cartilages from six healthy adult subjects (mean age of 53 years) were forensic waste from legal medicine with no need of informed consent after consultation with the national ethics committee and in strict agreement with French legislation. OA primary chondrocytes were cultured in Dulbecco’s modified Eagle’s medium (DMEM) containing 10% fetal calf serum as described [23]. Primary OA chondrocytes (2.5 × 10^5^ cells) were pelleted by centrifugation in 15-mL conical tubes, placed in three-dimensional (3D) setting for 7 days in chondrogenic medium—DMEM supplemented with 0.1 μM dexamethasone (Sigma-Aldrich, St. Louis, MO, USA), 1 mM pyruvate sodium (Invitrogen, Paisley, UK), 0.17 mM ascorbic acid (Sigma-Aldrich), 0.35 mM Proline (Sigma-Aldrich), 1% Insulin Transferin Selenium (Lonza, Basel, Switzerland), 2 mM L-glutamine (Lonza), 100 U/mL penicillin, and 100 μg/mL streptomycin (Lonza)—supplemented with transforming growth factor-beta 3 (TGF-β3) at 10 ng/mL (R&D Systems, Minneapolis, MN, USA). Treatment with recombinant human IL-1β at 10 ng/mL (R&D Systems) was applied for the first 5 days. Wild-type or *ink4a* knockout mice (1 month old) were obtained as reported [24]. Mice were housed and cared for in accordance with the laboratory animal care guidelines. Approval was obtained from the regional ethics committee on animal experimentation before initiation of the study (approval CEEA-LR-10042). Experiments were performed in accordance with the regional ethics committee on animal research and care.

### MicroRNA array analysis

Total RNA was extracted from chondrocytes in micropellet treated (or not) with IL-1β by using a miRvana isolation kit (Ambion, Carlsbad, CA, USA). MiRNA expression profiling was performed by using Miltenyi (Bergisch Gladbach, Germany) microarray facilities. Labeling and hybridization were performed in accordance with the protocol of the manufacturer. Raw data were normalized and additional data analysis was performed as described previously [25]. Microarray data are available in the ArrayExpress database [26] under accession number E-MTAB-2229.

### Reverse transcription, microRNA reverse transcription, and quantitative polymerase chain reaction

One microgram of Trizol-extracted total RNAs including microRNAs from the different samples were poly(A)-tailed with poly(A) polymerase (NEB M0276L). Then the polyadenylated RNA samples were reverse-transcribed as previously described using 50 units M-MLV Reverse Transcriptase (Invitrogen, Carlsbad, CA, USA) and either random primers or dTmiR adapter [27]. For microRNA and mRNA quantitative analysis, cDNA was mixed with Sybr Green Master Mix (Roche Diagnostics, Indianapolis, IN, USA) in 96-well plates containing specific primers for hsa-miR-24 (universal reverse + specific primer), interest genes or the ribosomal subunit protein-9, housekeeping gene (*hRSP9*). Quantitative polymerase chain reaction (qPCR) conditions as described [27] used the following primer-probe combinations: for hRSP9 sense 5′-GATTACATCCTGGGCCTGAA antisense 5′-ATGAAGGACGGGATGTTCAC; for Aggrecan (hACAN) sense 5′- TCGAGGACAGCGAGGCC anti-sense 5′-TCGAGGGTGTAGCGTGTAGAGA; for hCOL2A1, variant 2 (hCol2A1) sense 5′-CAGACGCTGGTGCTGCT anti-sense 5′-TCCTGGTTGCCGGA CAT; For hMMP13 sense 5′-TAAGGAGCATGGCGACTTCT anti-sense 5′-GTCTGGCGTTTTTGGATGTT; for hp16^INK4a^ sense 5′- GAAGGTCCCTCAGACATCCCC anti-sense 5′-CCCTGTAGGACCTTCGGTGAC; for hsa-miR-24 sense 5′-TGGCTCAGTTCAGCAGGAACAG Universal Reverse 5′- GCGAGCACAGAATTATACGACT.

### cDNA constructs and luciferase reporter assay

Plasmids encoding for miR-24-2 promoter (−2041 base-pair) Luciferase and CMV β-galactosidase were provided by Charles Lecellier [28]. Empty vector or p16^INK4a^ encoding vector were purchased from Addgene [29]. For promoter activity assay, OA human primary chondrocytes were transfected at day 0, placed in pellet culture conditions and treated with IL-1β during 48 hours. Cells were then lysed according to the dual luciferase/βgal kit (Promega, Charbonnières-les-Bains, France). Firefly Luciferase and β-galactosidase activities were detected using specific substrates with MultiScan FC (Thermo Scientific, Loughborough, UK). Luciferase activity was normalized to β-galactosidase activity.

### *In vitro* differentiation of human bone marrow-mesenchymal stromal cells to chondrocytes

Human bone marrow-mesenchymal stromal cell (hBM-MSC) culture were established from bone marrow of patients undergoing Hip replacement surgery, after patient informed written consent and approval by the local and national ethics committee (“Cellule de bioéthique de la direction générale pour la recherche et innovation, Ministère de l’Enseignement supérieur et de la Recherche”; registration number DC-2009-1052). Human mesenchymal stromal cells (hMSCs) were isolated and amplified by using a complete alpha-minimum essential medium supplemented with 10% fetal bovine serum + 1 ng/mL of basic fibroblast growth factor. hBM-MSCs were positive for CD44, CD73, CD90, and CD105 but negative for CD14, CD34, and CD45. Chondrogenic differentiation of BM-MSCs was induced by 21-day culture in micropellet [30]. Chondrogenesis was monitored by measuring the expression of chondrocyte-specific markers by reverse transcription-qPCR (RT-qPCR) as described [30].

### Transfections

Human chondrocytes (75 × 10^4^) were transfected with 15 μg of plasmid for 24 hours by using Transit-LT1 Reagent (Euromedex, Souffelweyersheim, France). Chondrocytes were transfected with 100 nM of AntagomiR control or AntagomiR-24 (purchased from Ambion) by using oligofectamine (Invitrogen, USA). After transfection, cells were trypsinized and pelleted in chondrogenic medium and cultured for 7 days.

### Western Blot and enzyme-linked immunosorbent assay

For Western blotting, chondrocytes in pellet cultures were lysed in RIPA-Benzonase buffer [22]. After the addition of the lysis buffer, the samples were left on ice for 15 minutes with vortexing every 5 minutes for 10 seconds. Lysate protein samples were then sonicated for 5 minutes, followed by centrifugation at 7000 *g* for 15 minutes. The protein quantity loaded on Western blot gel corresponded to 25 × 10^4^ cells. Primary antibodies and dilutions were anti-CDKN2A/p16INK4a (ab54210; Abcam; 1:1,000) and anti-β actin (Sigma-Aldrich A228; 1:8,000). Secondary antibody used for Western blot analysis was goat anti-mouse IgG HRP conjugate (Jackson ImmunoResearch Laboratories, Inc., West Grove, PA, USA; 115-035-003; 1:80,000). Enzyme-linked immunosorbent assays (ELISAs) were performed by using kits (IL-6, IL-8, pro-MMP13, and MMP1) from R&D Systems on supernatants stored at −20°C until analysis. Data were normalized and expressed as picograms per milliliter.

### Immunohistochemistry and staining

Samples were fixed in 3.7% paraformaldehyde for 24 hours, washed in phosphate-buffered saline (PBS), and processed for routine histology. Paraffin-embedded sample sections (5 μm) were rehydrated through a gradient of xylene and ethanol. Samples were first incubated for 20 minutes at room temperature with pepsin solution (EmergoEurope, The Hague, The Netherlands) for antigen retrieval. Endogenous peroxidase blocking was done with 1% H_2_O_2_ for 20 minutes at room temperature. Samples were pre-incubated with blocking solution (PBS + 10% goat serum + 0.1% Triton) for 30 minutes at room temperature. Endogenous biotins were blocked by using a Streptavidin/Biotin blocking kit (Vector SP-2002) for 30 minutes. Primary antibody anti-CDKN2A/p16^INK4a^ monoclonal mouse antibody (1:200; Abcam ab54210) was incubated for 72 hours at 4°C. Incubation with Biotin-coupled secondary antibody, IgG (1:200; ABC kit Vector PK6100) was done for 1 hour at room temperature. Incubation with Avidin/Biotin complex (ABC kit Vector PK6100) was done for 30 minutes at room temperature. Immunolocalized antigens were detected by means of a DAB revelation kit (Sigma-Aldrich). Safranin-O staining was performed as described [31]. Quantification of proliferating cell nuclear antigen (PCNA)-positive or PCNA-negative hypertrophic chondrocytes was performed on four different sections of long bones of four mice of each genotype by using ImageJ software (D-0426) in accordance with the instructions of the manufacturer.

### Statistical analysis

Experiments were performed with at least three independent individual chondrocyte or MSC samples. Comparisons of two conditions were done by using a paired Student *t* test. Unpaired Mann-Whitney test was applied for cartilage samples by using GraphPad Prism Software (GraphPad Software, Inc., La Jolla, CA, USA). Differences were considered significant when *P* values were less than 0.05.

## Results and discussion

### p16^INK4a^ accumulates with other senescence-associated secretory phenotype factors in interleukin-1-beta-treated mature chondrocytes

**Figure 1:**
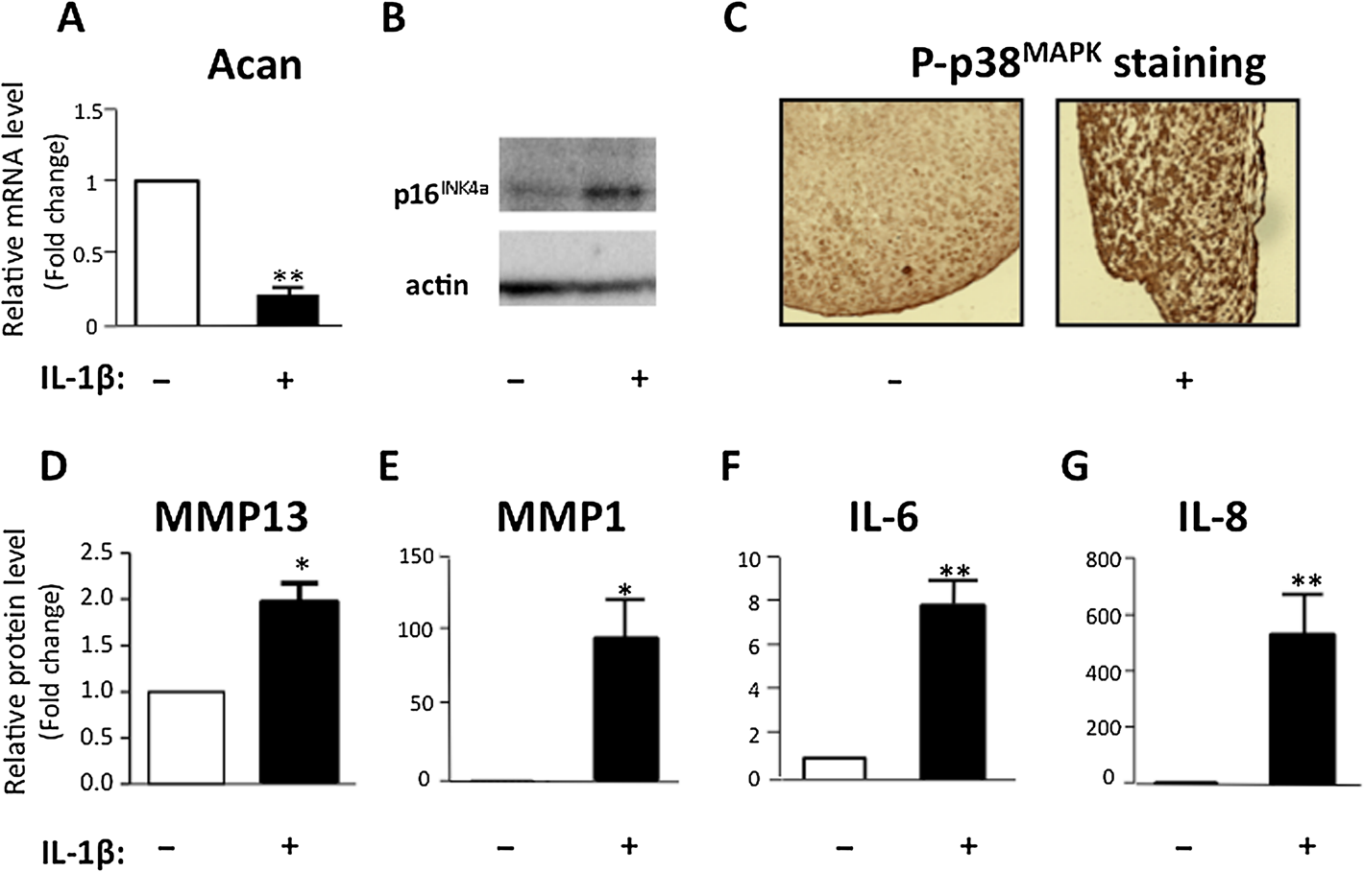
**Interleukin-1-beta (IL-1β) induces both p16**^**INK4a **^**expression and a senescence-associated secretory phenotype (SASP) in mature chondrocytes.** Osteoarthritis (OA) human primary chondrocytes were placed in pellet culture and treated with IL-1β (10 ng/mL) for 5 days. **(A)** Aggrecan mRNA (Acan) expression level was evaluated by reverse transcription-quantitative polymerase chain reaction (RT-qPCR) (n = 3). **(B)** p16^INK4a^ protein expression level was measured by Western blotting. **(C)** P-p38^MAPK^ protein level was detected by immunohistochemistry (IHC) on a section of paraffin-embedded pellets. Images were taken with a ×20 objective. **(D-G)** Matrix metalloprotease 1 (MMP1), MMP13, IL-6, and IL-8 secretion were measured by enzyme-linked immunosorbent assay (ELISA) (n = 4). Data are shown as mean ± standard deviation (SD) of fold changes compared with control. *P <0.05, **P <0.01.

We first wanted to establish an *in vitro* model based on mature chondrocytes, mimicking the senescence-like phenotypes found in OA cartilage. This model should associate a 3D setting reproducing that of chondrocytes within the tissue, expression of senescence markers such as p16^INK4a^, and production of SASP factors. We therefore isolated primary chondrocytes from OA cartilage patients and that were placed in pellet culture conditions to maintain/reinduce the chondrocyte phenotype. We used IL-1β as one of the major cytokine inducers of cartilage degradation in OA [32] to induce senescence-associated phenotype. We observed that, in response to chronic IL-1β treatment for 5 days, mature chondrocytes lose (as expected) the expression of specific differentiation markers such as Aggrecan (Figure 1A) [33], concomitantly with p16^INK4a^ accumulation (Figure 1B), phospho-active form of p38^MAPK^ (Figure 1C), and production of reactive oxygen species (data not shown), hallmarks of senescence-associated signaling pathways [34]. Furthermore, as expected, IL-1β-treated mature chondrocytes significantly produce senescence-associated secretory factors such as MMP1 and MMP13, two markers normally associated with chondrocyte terminal differentiation onset and IL-6 and IL-8, two pro-inflammatory cytokines (Figure 1D-G). Therefore, this 3D *in vitro* model recapitulates the p16^INK4a^-associated secretory phenotype characterizing senescent-like chondrocytes found in OA cartilage.

### The senescence marker p16^INK4a^ is expressed during *in vitro* chondrogenesis and participates in the terminal differentiation-dependent cell cycle exit during endochondral ossification

**Figure 2:**
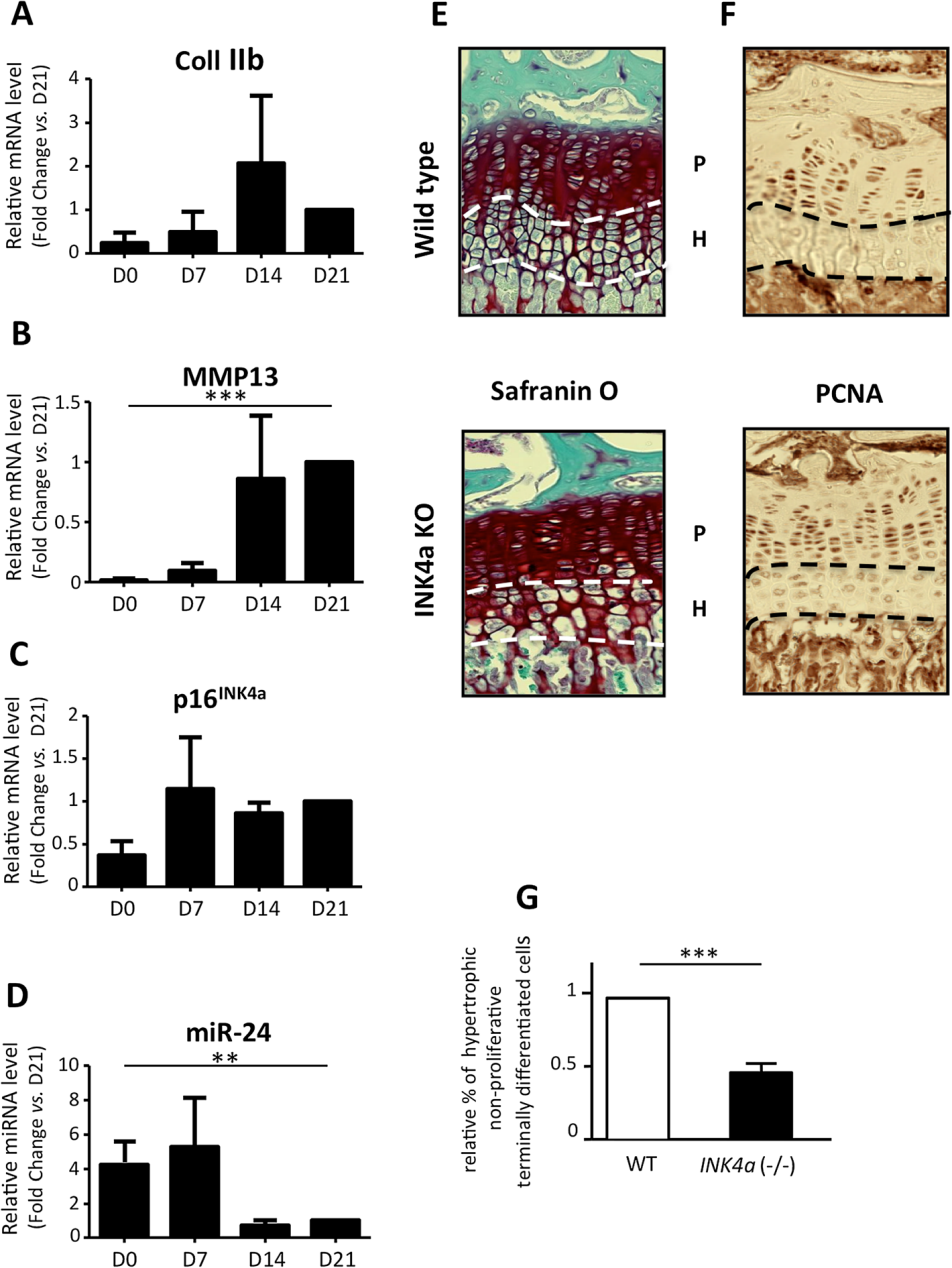
**MiR-24 and p16**^**INK4a **^**expressions during chondrogenesis and role of p16**^**INK4a **^**in chondrocyte cell cycle arrest.** Human primary mesenchymal stromal cells (MSCs) were placed in three-dimensional (3D) culture conditions for 21 days. RNAs were harvested at indicated time points. **(A-D)** Gene expression analysis was performed by reverse transcription-quantitative polymerase chain reaction (RT-qPCR) for Collagen 2b, matrix metalloprotease 13 (MMP13), p16^INK4a^, and miR-24. Data are shown as mean ± standard deviation (SD) (n = 3) normalized to D21. Immunohistochemistry (IHC) was performed on sections of formalin fixed paraffin-embedded long bones of transgenic mice deficient in p16^INK4a^ or wild-type at the age of 1 month.** (E,F)** Growth plate was marked by Safranin-O staining and proliferating cell nuclear antigen (PCNA) (P, proliferative zone; H, pre-hypertrophic/hypertrophic zone). Images were taken with ×20 objective. **(G)** Quantification of the percentage of hypertrophic non-proliferative terminally differentiated cells on total cells within the growth plate in transgenic and wild-type mice (n = 4) was carried out by using ImageJ software. Data were normalized to 1 for wild-type and are shown as mean ± SD. **P <0.01, ***P <0.001.

Besides inflammatory cytokines, aberrant chondrogenic differentiation signals can also promote OA features. For instance, Notch activation and HIF-2α transcription factor, both inducers of OA, are also central in controlling chondrocyte terminal differentiation onset during *in vitro* chondrogenesis and *in vivo* endochondral ossification [9,10]. Thus, we next wanted to determine whether p16^INK4a^ could also be part of a normal *in vitro* and *in vivo* chondrogenic differentiation process as has been reported for Notch or HIF-2α. We therefore evaluated the p16^INK4a^ expression profile in human MSCs from three different donors undergoing TGF-β3-induced chondrogenic differentiation to recapitulate *in vitro* all stages of chondrogenesis. Surprisingly, p16^INK4a^ expression increases during chondrogenesis in parallel with a chondrocyte differentiation marker such as collagen IIB at days 7 to 14 and in a concomitant manner with MMP13, a chondrocyte terminal differentiation marker, at day 21 (Figure 2A-D). Of note, during chondrogenesis, p14^ARF^ mRNA, an alternative splicing form of p16^INK4a^, was not detected at any time (data not shown). Thus, the senescence marker p16^INK4a^, which is known to be required for astrocyte [4] or epidermal cell [5] differentiation, seems also to play a role during chondrogenesis. To dissect its *in vivo* function during chondrogenic differentiation, we compared the endochondral ossification process of transgenic mice deficient in *ink4a* locus with that of wild-type mice. PCNA and Safranin-O staining were used to quantify proliferative chondrocytes versus non-proliferative chondrocytes within the growth plate of 1-month-old mice. Without affecting the total number of chondrocytes, *ink4a* ablation reduces the height of hypertrophic terminally differentiated non-proliferative chondrocytes by 48% ± 2.2 (Figure 2E-G), demonstrating a role for *ink4a* locus in chondrocyte cell fate decision to engage in terminal differentiation. A main function of p16^INK4a^ is to specifically inhibit cell cycle progression by targeting G1 CDK4/6 activities [35] and therefore maintain retinoblastoma (pRb), p107, and p130 under their active unphosphorylated forms [36]. These three pocket proteins are known to control chondrocyte cell fate decision during bone growth [37-39]. Interestingly, two other CKIs—p27^KIP1^ and p57^KIP2^—are also part of the cell cycle regulation during terminal differentiation through CDK inhibition leading to pocket proteins activation [40,41]. But only p16^INK4a^ plays a major role in both cellular senescence and differentiation onset as revealed in *ink4a* knockout mice studies. Taken together, these findings point at the multiple roles of several CKIs and their indirect targets—pRb, p107, and p130—as orchestrators of the differentiation program and, in particular, in chondrocyte terminal differentiation during bone development.

### Expression of p16^INK4a^ is sufficient for MMP1 and MMP13 secretion by mature chondrocytes

**Figure 3:**
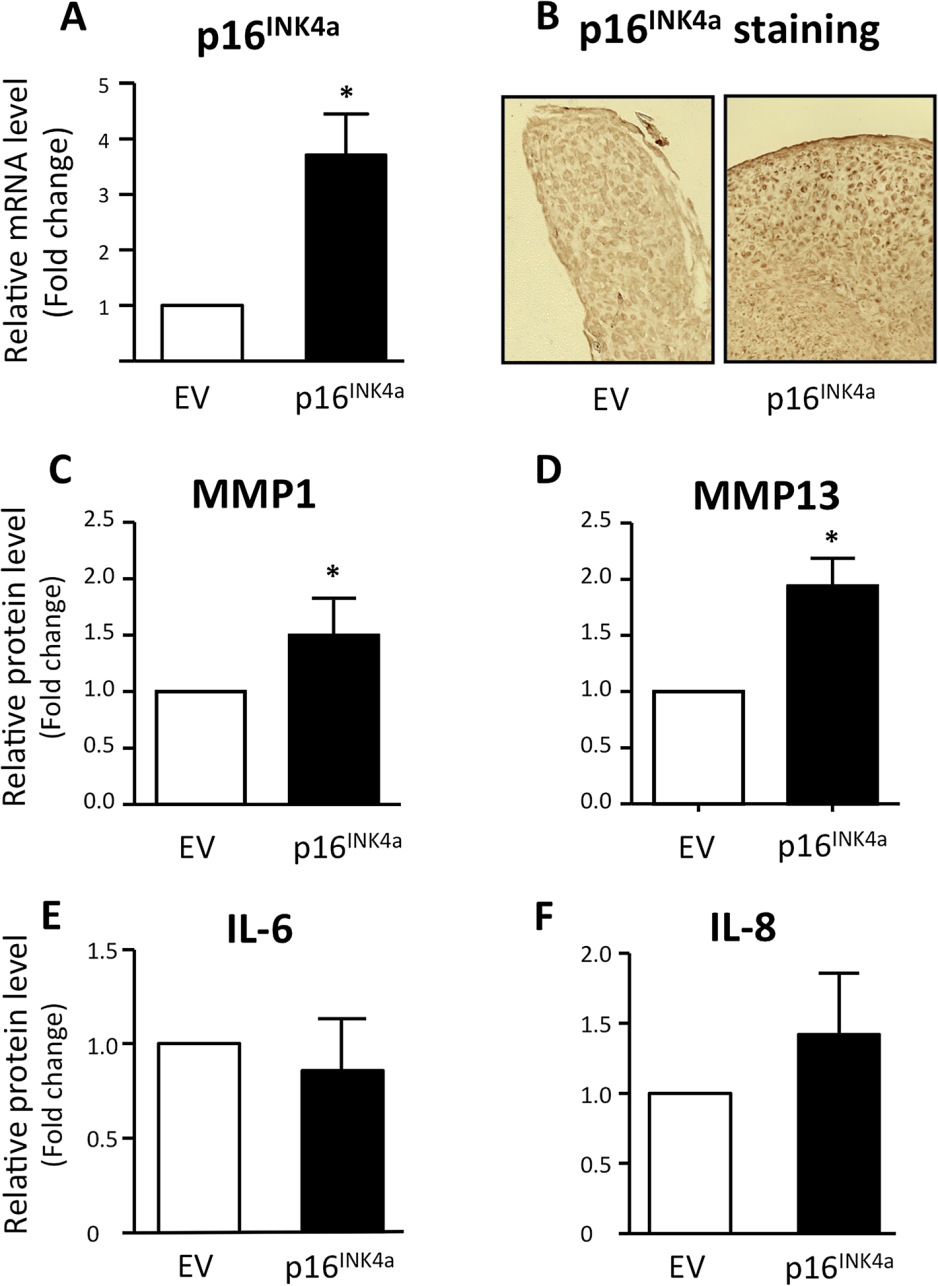
**Cyclin-dependent kinase inhibitor p16**^**INK4a **^**participates in matrix metalloproteinase 1 (MMP1) and MMP13 expression in mature chondrocytes.** Osteoarthritis (OA) human primary chondrocytes were transfected with vector encoding for p16^INK4a^ or empty vector (EV) for 24 hours and placed in pellet for 7 days. **(A)** Gene expression analysis was performed by reverse transcription-quantitative polymerase chain reaction (RT-qPCR) for p16^INK4a^ (n = 5). **(B)** p16^INK4a^ protein expression was detected by immunohistochemistry (IHC) on pellet paraffin sections. Images were taken with a ×20 objective. **(C-F)** MMP1, MMP13, interleukin-6 (IL-6), and IL-8 secretions were measured by enzyme-linked immunosorbent assay (ELISA) (n = 5). Data are shown as mean ± standard deviation (SD). *P <0.05.

To determine how Ink4a could participate in both OA initiation and chondrocyte terminal differentiation, we assessed whether p16^INK4a^ expression impacts the establishment of matrix remodeling secretome common in both events. We thus transiently transfected a p16^INK4a^-encoding vector in human chondrocytes, before initiating the pellet culture for 7 days and in the absence of IL-1β. Overexpression of p16^INK4a^ was checked at the mRNA level (Figure 3A) and protein level (Figure 3B). Compared with the control, p16^INK4a^-overexpressing mature chondrocytes produced significantly higher levels of MMP1 and MMP13 but did not modulate IL-6 and IL-8. Of note, our findings confirm recent published data showing that p16^INK4a^ is dispensable for the establishment of inflammatory secretome associated with senescent fibroblasts [42]. Taken together, our results revealed that the senescence-associated CKI, p16^INK4a^, triggers the secretion of both MMP1 and MMP13 in mature chondrocytes. These two metalloproteases are matrix remodeling enzyme family members playing a central role in physiological and pathological processes occurring in cartilage [33,43,44]. Indeed, both are expressed during chondrocyte terminal differentiation within the growth plate to coordinate matrix remodeling that promotes bone growth [12] but also in OA articular cartilage during disease progression [45]. How could p16^INK4a^ control MMPs production? Firstly, ink4a locus could directly regulate MMP1 transcriptional activation through its described physical interaction with APA1/ZNF410 transcription factor which is bound to MMP1 promoter [46]. Secondly, by activating pRb-, p130-, and p107-dependent terminal differentiation programs through CDK inhibition, p16^INK4a^ accumulation could also contribute indirectly to MMPs production in OA and during endochondral ossification. Indeed, these three pocket proteins can modulate gene expression and cell fate decision through their interactions with chromatin-modifying enzymes [47]. One found, among these enzymes, histone deacetylase (HDAC) family members, which are controlling chondrocyte pre-hypertrophy/hypertrophy transition [16,17]. Remarkably, recent work from Culley and colleagues [48] reveals that pharmacological inhibition of HDACs prevents the expression of metalloproteases such as MMP13 by OA chondrocytes. Thus, p16^INK4a^ contributes to MMP secretion by activating the pocket protein-HDAC axis in pathological and physiological conditions.

### p16^INK4a^ induction correlates with miR-24 repression in interleukin-1-beta-treated chondrocytes, osteoarthritic cartilage, and the end of an *in vitro* chondrogenesis

**Figure 4:**
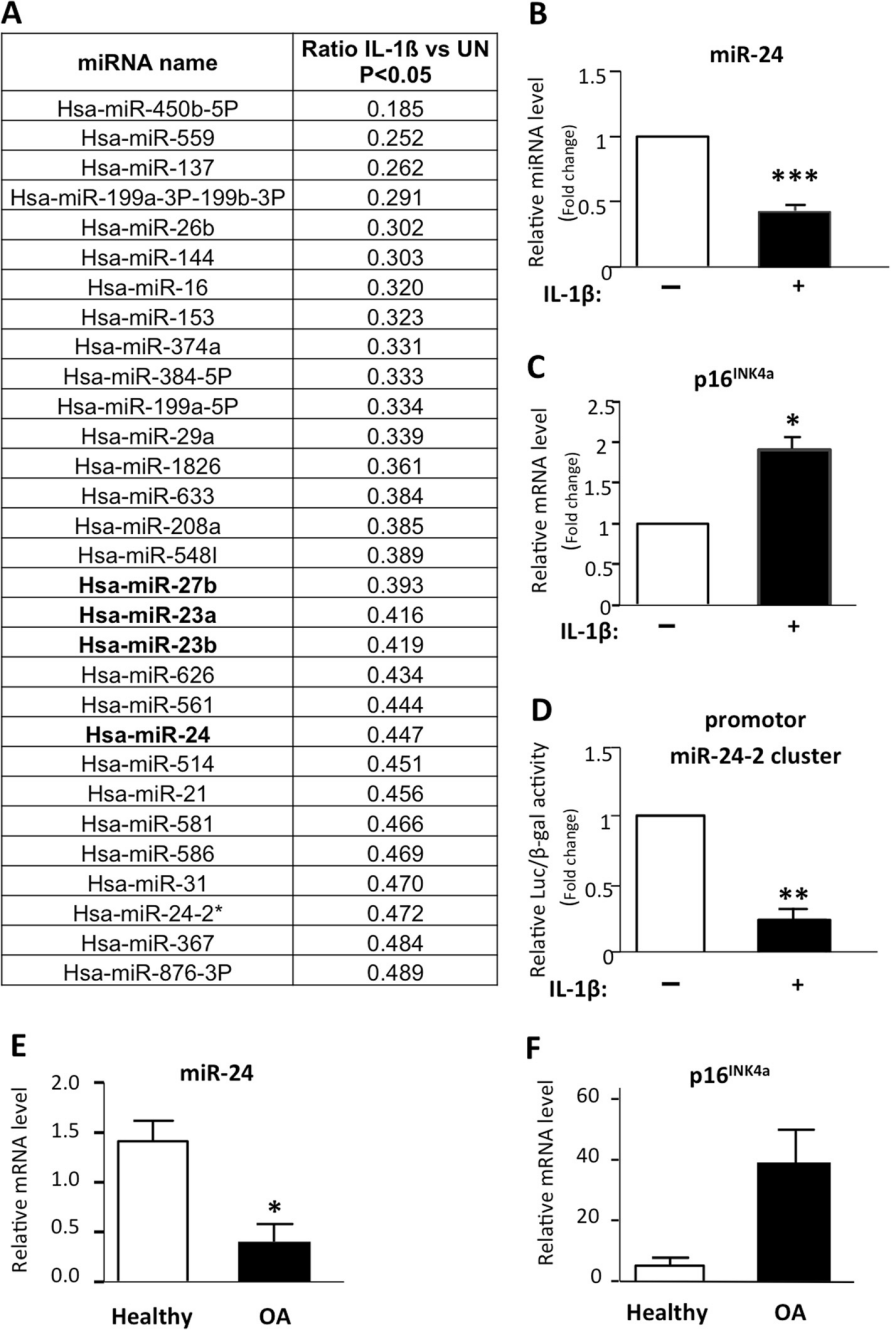
**MiR-24 repression correlates with p16**^**INK4a **^**induction in interleukin-1-beta (IL-1β)-treated chondrocytes and osteoarthritis (OA) cartilage.** OA human primary chondrocytes are placed in pellet and treated with IL-1β at 10 ng/mL for 5 days. **(A)** List of microRNAs downregulated by IL-1β. **(B,C)** Gene expression analysis was performed by reverse transcription-quantitative polymerase chain reaction (RT-qPCR) for miR-24 and p16^INK4a^ (n = 3). **(D)** Luciferase/β-gal activities of promoter miR-24-2 cluster after IL-1β treatment (n = 3). Data are shown as mean ± standard deviation (SD). MiR-24 repression in OA cartilage samples is shown. **(E,F)** MiR-24 repression in OA cartilage: gene expression for miR-24 and p16^INK4a^ on OA (n = 5) and healthy (n = 6) cartilage samples. *P <0.05, **P <0.01, ***P <0.001.

We next wanted to identify putative regulators of p16^INK4a^ in mature chondrocytes. Since miRs have been shown to play an important role in cartilage physio-pathology [16], we asked whether some miRs could regulate p16^INK4a^ expression in IL1β-treated chondrocytes. miR-array analysis was performed on small RNAs extracted from chondrocytes that were from three different donors and that were cultured in pellets in the presence or absence of IL-1β (Figure 4). Bioinformatic analysis revealed that 179 miRs (128 up and 51 down) are differentially expressed in response to IL-1β compared with untreated cells. These deregulated miRs have at least a 1.4-fold change and a *P* value of less than 0.05 (Figure 4A and data not shown). Interestingly, we found among them several previously OA-associated miRs such as miR-27b, miR-199, miR-29a, miR-26, and miR-365 [16,17].

Since inhibition of miR-processing enzymes such as Dicer induces senescence-associated phenotypes in primary cells [49] and promotes chondrocyte terminal differentiation in animal models [50], we focused our attention on miRs that were repressed by IL-1β (Figure 4A). We found miR-24, a known negative regulator of p16^INK4a^, through the presence of two binding sites for this miR within its encoding and 3′ untranslated region (UTR) [51]. We next confirmed, on three independent chondrocyte samples placed in 3D, that IL-1β significantly repressed miR-24 expression (Figure 4B) with a concomitant expected induction of p16^INK4a^ mRNA (Figure 4C).

miR-24 is encoded by two genes: miR-24-1 and miR-24-2 [52]. These genes are organized in a cluster including three different miRs (miR-23a or b/27a or b/24). Each cluster is regulated by one promoter common for the three miRs of the cluster [52]. Our array analysis revealed that, upon IL-1β stimulation, chondrocytes show a reduced expression of several members of these two clusters (Figure 4A), suggesting a global repression of the transcription of the clusters. In keeping with this hypothesis, we confirmed the transcriptional repression of miR-24-2 promoter, upon IL-1β addition (Figure 4D), by using a reporter luciferase assay (Figure 4D) previously described [28].

An increase in expression of p16^INK4a^ has been demonstrated in cartilage from patients with OA [13]. We then checked whether miR-24 expression could be reversely correlated with that of p16^INK4a^ in OA cartilage compared with healthy cartilage. By RT-qPCR on mRNA from OA (n = 5) versus healthy (n = 6) human cartilage samples, we revealed a significant miR-24 downregulation in OA cartilage (Figure 4E) while p16^INK4a^ is increased (Figure 4F). These results were confirmed at the protein level on serial sections of OA cartilage samples by using p16^INK4a^ immunohistochemistry and miR-24 *in situ* hybridization (Additional file 1).

Figure 2 shows that p16^INK4a^ mRNA accumulates throughout the time course of an *in vitro* chondrogenesis from days 7 to 21 (Figure 2C). We therefore evaluated, during chondrogenesis, whether the expression of miR-24 could also be reciprocal to that of p16^INK4a^ expression. By RT-qPCR, we revealed that, compared with days 0 to 7, miR-24 level is decreased at day 14 and significantly at day 21 (Figure 2D) in parallel with an increase in expression of the terminal differentiation marker, MMP13 (Figure 2B), while p16^INK4a^ remains elevated (Figure 2C). Taken together, these results demonstrate that the expressions of Ink4a and its epigenetic regulator are mutually exclusive in both *in vitro* and *in vivo* OA models and during the end of the chondrogenesis. Moreover, miR-24 downregulation seems to follow and sustain a high level of p16^INK4a^ rather than initiate p16^INK4a^ accumulation.

### MiR-24 downregulation is sufficient to trigger p16^INK4a^ expression and MMP1 production in mature chondrocytes

**Figure 5:**
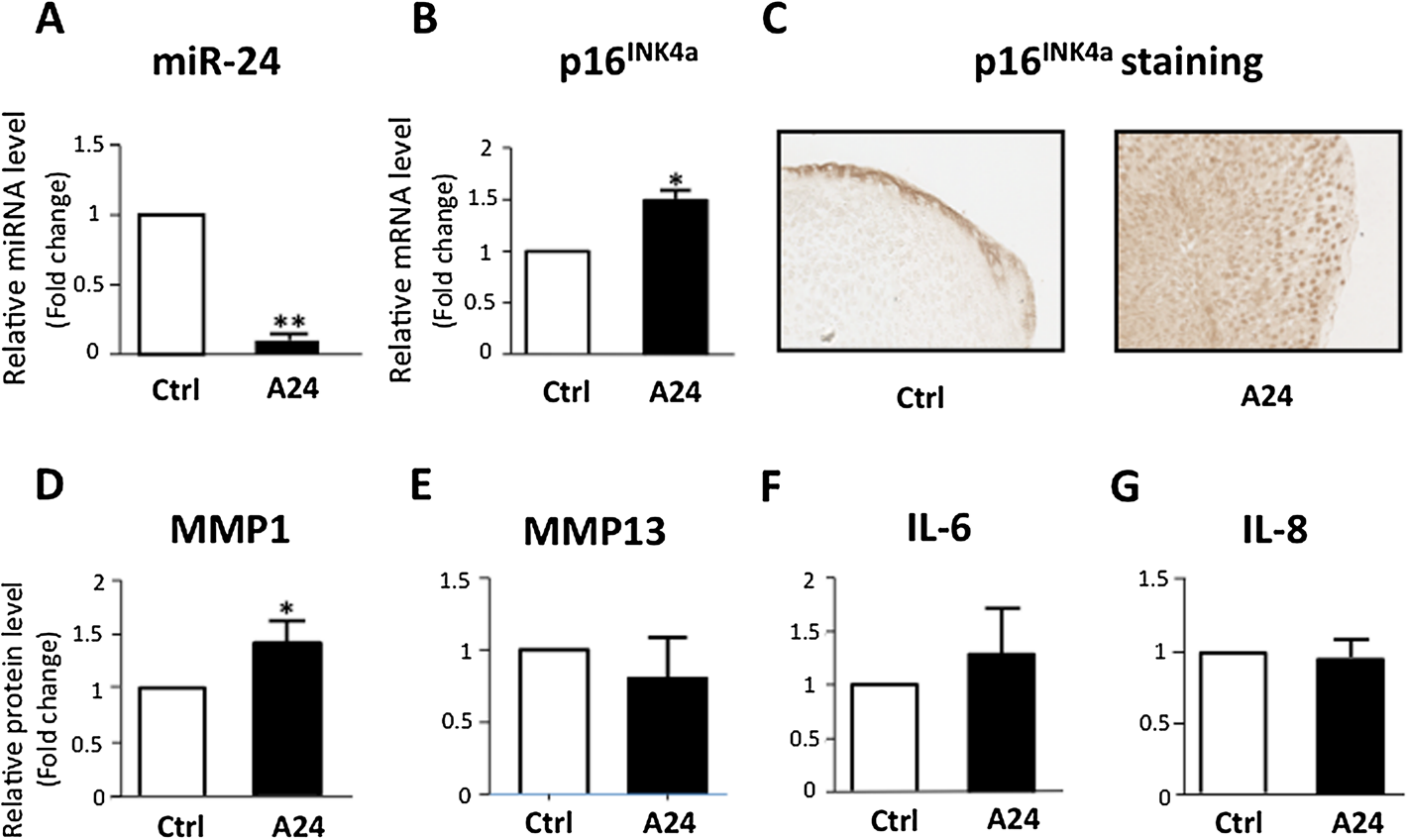
**MiR-24 downregulation is sufficient to trigger p16**^**INK4a **^**expression and matrix metalloproteinase 1 (MMP1) production in mature chondrocytes.** Osteoarthritis (OA) human primary chondrocytes were transfected with irrelevant antagomiR (Ctrl) and antagomiR-24 (A24) at a concentration of 100 nM. They were placed in pellet culture conditions for 7 days. **(A,B)** Gene expression analysis was performed by reverse transcription-quantitative polymerase chain reaction (RT-qPCR) for miR-24 and p16^INK4a^ (n = 4). **(C)** p16^INK4a^ protein expression by immunohistochemistry (IHC) on pellet paraffin-embedded sections. Images were taken with a ×20 objective. **(D-G)** MMP1, MMP13, interleukin-6 (IL-6), and IL-8 secretion were measured by enzyme-linked immunosorbent assay (ELISA) (n = 4). Data are shown as mean ± standard deviation (SD). *P <0.05.

Finally, we aimed at determining whether modulation of miR-24 could be sufficient to promote p16^INK4a^ accumulation and p16^INK4a^-dependent matrix remodeling secretome by using our 3D chondrocyte model. Because miR-24 overexpression has been reported to induce apoptosis by repressing DNA damage response pathways [53], we relied on a loss-of-function experiment based on transfection of chondrocytes by either a specific inhibitor of miR-24 (antagomiR-24) or an irrelevant antagomiR as control. As expected, transfection of antagomiR-24 led to a dramatic downregulation of miR-24 level (Figure 5A) and the concomitant significant upregulation of p16^INK4a^ at both mRNA (Figure 5B) and protein levels as shown by immunostaining with p16^INK4a^ antibodies (Figure 5C). Furthermore, we showed that miR-24 downregulation is sufficient to promote a marked increase in MMP1 secretion (Figure 5D) but has no significant effect on MMP13, IL-6, or IL-8 secretion (Figure 5E-G), suggesting the existence of a direct axis miR-24-p16^INK4a^-MMP1. The discrepancy between this result and p16^INK4a^ overexpression alone could be explained by mutual redundancy and interference between the signaling pathways. Nevertheless, miR-24 is part of a cluster containing two other miRs and regulated by the same promoter (Figure 4D). Therefore, miR-24 repression is *in vivo* always accompanied by that of miR27a/b and miR23a/b (Figure 4A and D). Remarkably, miR27b was recently shown to inhibit MMP13 expression in IL-1β-treated chondrocytes [21] and miR23a/b could negatively regulate Runx2, a transcription factor involved in chondrocyte terminal differentiation, OA, and osteoblastogenesis [54]. Thus, repression of these clusters during OA progression within articular cartilage would promote the appearance of several OA-induced features, including p16^INK4a^, MMP1, MMP13, but also Runx2. These findings propose that miR-24-1/miR-24-2 clusters, together with the recently identified miR-140, which targets ADAMTS5 and HDAC4, two hypertrophic inducers [19,20], are crucial in preventing chondrocyte terminal differentiation in OA.

## Conclusions

Determining the role and the regulatory pathways controlling p16^INK4a^ expression in chondrocytes during OA progression is essential for future innovative long-term therapeutic approaches. In the present work, we demonstrated that the senescence CKI, p16^INK4A^, is also associated with chondrocyte terminal differentiation and can regulate the expression of matrix remodeling metalloproteases MMP1 and MMP13. We further showed that miR-24 expression plays a role as a negative regulator of the p16^INK4a^/MMP1 axis.

We propose that—during OA progression, in response to IL-1β, or during endochondral-induced terminal chondrogenesis—a repression of miR-24- and miR-24-encoding clusters takes place. This is likely to trigger p16^INK4a^, MMP1, MMP13, and Runx2 expression, thereby pushing chondrocytes toward a senescent-like phenotype resembling that of terminally differentiated chondrocytes [7]. Accumulation of p16^INK4a^-positive chondrocytes within articular cartilage could thus be deleterious not only for tissue regeneration by blocking cell proliferation and replacement but also for tissue integrity through MMPs secretion [7,55-57]. On the other hand, p16^INK4a^ accumulation within the growth plate will favor bone development. One therapeutic strategy for OA treatment could be to restore/maintain the expression level of miR-24-encoding clusters in order to prevent p16^INK4a^-dependent pathways in articular chondrocytes.

## Abbreviations

3D: three-dimensional; BM-MSC: bone marrow-mesenchymal stromal cell; CDK: cyclin-dependent kinase; CKI: cyclin-dependent kinase inhibitor; DMEM: Dulbecco’s modified Eagle’s medium; hBM-MSC: human bone marrow-mesenchymal stromal cell; HDAC: histone deacetylase; HIF-2α: hypoxy-inducible factor-2; IL: interleukin; miR: microRNA; MMP: matrix metalloproteinase; OA: osteoarthritis; PBS: phosphate-buffered saline; PCNA: proliferating cell nuclear antigen; pRb: retinoblastoma protein; RT-qPCR: reverse transcription-quantitative polymerase chain reaction; SASP: senescence-associated secretory phenotype; TGF-β3: transforming growth factor-beta 3.

## Competing interests

The authors declare that they have no competing interests.

## Authors’ contributions

DPh participated in immunochemistry, immunoassays, gene expression, statistical analysis and manuscript drafting. DG participated in MSC isolation, chondrogenesis experiments, and statistical analysis and critically revised the manuscript. DPl and EO participated in chondrocyte isolation, Western blot experiments, and manuscript drafting. FE participated in transfection experiments and immunoassay experiments and critically revised the manuscript. AD collected post-mortem human cartilages, participated in microRNA isolation, and critically revised the manuscript. Y-MP collected human OA cartilage, acquired clinical data, obtained patient written consents, and critically revised the manuscript. PC performed microarray analysis and participated in data interpretation, manuscript drafting, and critical reading. JP participated in mice INK4a KO design experiments and immunostaining knee analysis and critically revised the manuscript. CJ initiated the project; participated in experimental designs, microarray and data interpretation, and manuscript drafting; and critically revised the manuscript. DN participated in experimental conception, cell banking, data interpretation, and manuscript drafting and critical reading. RMB participated in experimental designs, microRNA isolation, Western blot analysis, data interpretation, and manuscript drafting and critical reading. J-MB participated in immunoassay experiments, collecting data, statistical analysis, manuscript drafting, and correspondence to editor. All authors read and approved the final manuscript.

## Supplementary Material

Additional file 1 **p16^INK4a^ and miR-24 are reversely correlated in osteoarthritis (OA) articular cartilage. (A)** p16^INK4a^ immunohistochemistry (IHC) on a cryosection of OA cartilage comprising superficial and intermediate layer. **(B)** miR-24 *in situ* hybridization on an adjacent section of the same OA cartilage sample. Results are representative of experiments on two OA cartilage samples. Chondrocytes expressing either p16^INK4a^ or miR-24 are marked by arrows showing mutual exclusion. OA cartilage samples were fixed with 4% paraformaldehyde during 3 hours at 4°C. After fixation, samples were placed in PBS with sucrose 30% during 24 hours at 4°C. The next day, PBS-sucrose 30% was replaced with Tek OCT solution and the samples were stored at −80°C. OA cartilage samples were sectioned at 13 μm and collected on Superfrost PLUS slides. *In situ* hybridization experiment was performed as described [58]. LNA DIG-hsa-miR-24 probe and DIG-has-miR-141c (as negative control) were purchased from Exiquon (Copenhagen, Denmark) and diluted at 1pM. Alkaline phosphatase conjugated anti-DIG- antibody was diluted at 1:2,000 in blocking solution. This file can be viewed with Acrobat Reader. Click here for file

## References

[B1] Zindy F Quelle DE Roussel MF Sherr CJ Expression of the p16INK4a tumor suppressor versus other INK4 family members during mouse development and aging Oncogene 1997 15 203 211 10.1038/sj.onc.1201178 9244355

[B2] Coppé J Desprez P Krtolica A Campisi J The senescence-associated secretory phenotype: the dark side of tumor suppression Annu Rev Pathol 2010 5 99 118 10.1146/annurev-pathol-121808-102144 20078217 PMC4166495

[B3] Baker DJ Wijshake T Tchkonia T LeBrasseur NK Childs BG van de Sluis B Kirkland JL van Deursen JM Clearance of p16Ink4a-positive senescent cells delays ageing-associated disorders Nature 2011 479 232 236 10.1038/nature10600 22048312 PMC3468323

[B4] Bachoo RM Maher EA Ligon KL Sharpless NE Chan SS You MJ Tang Y DeFrances J Stover E Weissleder R Rowitch DH Louis DN DePinho RA Epidermal growth factor receptor and Ink4a/Arf: convergent mechanisms governing terminal differentiation and transformation along the neural stem cell to astrocyte axis Cancer Cell 2002 1 269 277 10.1016/S1535-6108(02)00046-6 12086863

[B5] Paramio JM Segrelles C Ruiz S Martin-Caballero J Page A Martinez J Serrano M Jorcano JL The ink4a/arf tumor suppressors cooperate with p21cip1/waf in the processes of mouse epidermal differentiation, senescence, and carcinogenesis J Biol Chem 2001 276 44203 44211 10.1074/jbc.M105650200 11551927

[B6] Aigner T Söder S Gebhard PM McAlinden A Haag J Mechanisms of disease: role of chondrocytes in the pathogenesis of osteoarthritis–structure, chaos and senescence Nat Clin Pract Rheumatol 2007 3 391 399 10.1038/ncprheum0534 17599073

[B7] Loeser RF Aging and osteoarthritis: the role of chondrocyte senescence and aging changes in the cartilage matrix Osteoarthritis Cartilage 2009 17 971 979 10.1016/j.joca.2009.03.002 19303469 PMC2713363

[B8] Martin JA Buckwalter JA The role of chondrocyte senescence in the pathogenesis of osteoarthritis and in limiting cartilage repair J Bone Joint Surg Am 2003 85-A 106 110 12721352 10.2106/00004623-200300002-00014

[B9] Saito T Fukai A Mabuchi A Ikeda T Yano F Ohba S Nishida N Akune T Yoshimura N Nakagawa T Nakamura K Tokunaga K Chung UI Kawaguchi H Transcriptional regulation of endochondral ossification by HIF-2alpha during skeletal growth and osteoarthritis development Nat Med 2010 16 678 686 10.1038/nm.2146 20495570

[B10] Hosaka Y Saito T Sugita S Hikata T Kobayashi H Fukai A Taniguchi Y Hirata M Akiyama H Chung UI Kawaguchi H Notch signaling in chondrocytes modulates endochondral ossification and osteoarthritis development Proc Natl Acad Sci U S A 2013 110 1875 1880 10.1073/pnas.1207458110 23319657 PMC3562777

[B11] Goldring MB Otero M Inflammation in osteoarthritis Curr Opin Rheumatol 2011 23 471 478 10.1097/BOR.0b013e328349c2b1 21788902 PMC3937875

[B12] Tchetina EV Developmental mechanisms in articular cartilage degradation in osteoarthritis Arthritis 2011 2011 683970 22046522 10.1155/2011/683970 PMC3199933

[B13] Zhou HW Lou SQ Zhang K Recovery of function in osteoarthritic chondrocytes induced by p16INK4a-specific siRNA in vitro Rheumatology (Oxford) 2004 43 555 568 10.1093/rheumatology/keh127 15026580

[B14] Krol J Loedige I Filipowicz W The widespread regulation of microRNA biogenesis, function and decay Nat Rev Genet 2010 11 597 610 20661255 10.1038/nrg2843

[B15] Ebert MS Sharp PA Roles for microRNAs in conferring robustness to biological processes Cell 2012 149 515 524 10.1016/j.cell.2012.04.005 22541426 PMC3351105

[B16] Goldring MB Marcu KB Epigenomic and microRNA-mediated regulation in cartilage development, homeostasis, and osteoarthritis Trends Mol Med 2012 18 109 118 10.1016/j.molmed.2011.11.005 22178468 PMC3282171

[B17] Barter MJ Young DA Epigenetic mechanisms and non-coding RNAs in osteoarthritis Curr Rheumatol Rep 2013 15 353 23888362 10.1007/s11926-013-0353-z

[B18] Iliopoulos D Malizos KN Oikonomou P Tsezou A Integrative microRNA and proteomic approaches identify novel osteoarthritis genes and their collaborative metabolic and inflammatory networks PLoS One 2008 3 e3740 10.1371/journal.pone.0003740 19011694 PMC2582945

[B19] Tuddenham L Wheeler G Ntounia-Fousara S Waters J Hajihosseini MK Clark I Dalmay T The cartilage specific microRNA-140 targets histone deacetylase 4 in mouse cells FEBS Lett 2006 580 4214 4217 10.1016/j.febslet.2006.06.080 16828749

[B20] Miyaki S Nakasa T Otsuki S Grogan S Higashiyama R Inoue A Kato Y Sato T Lotz M Asahara H MicroRNA-140 is expressed in differentiated human articular chondrocytes and modulates interleukin-1 responses Arthritis Rheum 2009 60 2723 2730 10.1002/art.24745 19714579 PMC2806094

[B21] Akhtar N Rasheed Z Ramamurthy S Anbazhagan A Voss F Haqqi T MicroRNA-27b regulates the expression of matrix metalloproteinase 13 in human osteoarthritis chondrocytes Arthritis Rheum 2010 62 1361 1371 20131257 10.1002/art.27329 PMC3139404

[B22] Olivotto E Vitellozzi R Fernandez P Falcieri E Battistelli M Burattini S Facchini A Flamigni F Santi S Borzi' RM Chondrocyte hypertrophy and apoptosis induced by GROalpha require three-dimensional interaction with the extracellular matrix and a co-receptor role of chondroitin sulfate and are associated with the mitochondrial splicing variant of cathepsin B J Cell Physiol 2007 210 417 427 10.1002/jcp.20864 17096385

[B23] Battistelli M Borzì RM Olivotto E Vitellozzi R Burattini S Facchini A Falcieri E Cell and matrix morpho-functional analysis in chondrocyte micromasses Microsc Res Tech 2005 67 286 295 16173090 10.1002/jemt.20210

[B24] Serrano M Lee H Chin L Cordon-Cardo C Beach D DePinho RA Role of the INK4a locus in tumor suppression and cell mortality Cell 1996 85 27 37 10.1016/S0092-8674(00)81079-X 8620534

[B25] Chuchana P Holzmuller P Vezilier F Berthier D Chantal I Severac D Lemesre JL Cuny G Nirdé P Bucheton B Intertwining threshold settings, biological data and database knowledge to optimize the selection of differentially expressed genes from microarray PLoS One 2010 5 e13518 10.1371/journal.pone.0013518 20976008 PMC2958130

[B26] ArrayExpress - functional genomics data http://www.ebi.ac.uk/arrayexpress

[B27] Guérit D Philipot D Chuchana P Toupet K Brondello JM Mathieu M Jorgensen C Noël D Sox9-regulated miRNA-574-3p inhibits chondrogenic differentiation of mesenchymal stem cells PLoS One 2013 8 e62582 10.1371/journal.pone.0062582 23626837 PMC3633883

[B28] Saumet A Vetter G Bouttier M Portales-Casamar E Wasserman WW Maurin T Mari B Barbry P Vallar L Friederich E Arar K Cassinat B Chomienne C Lecellier CH Transcriptional repression of microRNA genes by PML-RARA increases expression of key cancer proteins in acute promyelocytic leukemia Blood 2009 113 412 421 18941112 10.1182/blood-2008-05-158139

[B29] Campeau E Ruhl VE Rodier F Smith CL Rahmberg BL Fuss JO Campisi J Yaswen P Cooper PK Kaufman PD A versatile viral system for expression and depletion of proteins in mammalian cells PLoS One 2009 4 e6529 10.1371/journal.pone.0006529 19657394 PMC2717805

[B30] Djouad F Delorme B Maurice M Bony C Apparailly F Louis-Plence P Canovas F Charbord P Noël D Jorgensen C Microenvironmental changes during differentiation of mesenchymal stem cells towards chondrocytes Arthritis Res Ther 2007 9 R33 10.1186/ar2153 17391539 PMC1906811

[B31] van Osch GJ van der Kraan PM van den Berg WB Site-specific cartilage changes in murine degenerative knee joint disease induced by iodoacetate and collagenase J Orthop Res 1994 12 168 175 10.1002/jor.1100120204 8164088

[B32] van de Loo FA Joosten LA van Lent PL Arntz OJ van den Berg WB Role of interleukin-1, tumor necrosis factor alpha, and interleukin-6 in cartilage proteoglycan metabolism and destruction, Effect of in situ blocking in murine antigen- and zymosan-induced arthritis Arthritis Rheum 1995 38 164 172 10.1002/art.1780380204 7848306

[B33] Goldring MB Otero M Plumb DA Dragomir C Favero M El Hachem K Hashimoto K Roach HI Olivotto E Borzì RM Marcu KB Roles of inflammatory and anabolic cytokines in cartilage metabolism: signals and multiple effectors converge upon MMP-13 regulation in osteoarthritis Eur Cell Mater 2011 21 202 220 21351054 10.22203/ecm.v021a16 PMC3937960

[B34] Campisi J Andersen JK Kapahi P Melov S Cellular senescence: a link between cancer and age-related degenerative disease? Semin Cancer Biol 2011 21 354 359 21925603 10.1016/j.semcancer.2011.09.001 PMC3230665

[B35] Sharpless NE Ink4a/Arf links senescence and aging Exp Gerontol 2004 39 1751 1759 10.1016/j.exger.2004.06.025 15582292

[B36] Ashizawa S Nishizawa H Yamada M Higashi H Kondo T Ozawa H Kakita A Hatakeyama M Collective inhibition of pRB family proteins by phosphorylation in cells with p16INK4a loss or cyclin E overexpression J Biol Chem 2001 276 11362 11370 10.1074/jbc.M007992200 11152455

[B37] Miller JP Yeh N Vidal A Koff A Interweaving the cell cycle machinery with cell differentiation Cell Cycle 2007 6 2932 2938 10.4161/cc.6.23.5042 18000404

[B38] LuValle P Beier F Cell cycle control in growth plate chondrocytes Front Biosci 2000 5 D493 D503 10.2741/LuValle 10799356

[B39] Yeh N Miller JP Gaur T Capellini TD Nikolich-Zugich J de la Hoz C Selleri L Bromage TG van Wijnen AJ Stein GS Lian JB Vidal A Koff A Cooperation between p27 and p107 during endochondral ossification suggests a genetic pathway controlled by p27 and p130 Mol Cell Biol 2007 27 5161 5171 10.1128/MCB.02431-06 17502351 PMC1951950

[B40] Kiyokawa H Kineman RD Manova-Todorova KO Soares VC Hoffman ES Ono M Khanam D Hayday AC Frohman LA Koff A Enhanced growth of mice lacking the cyclin-dependent kinase inhibitor function of p27(Kip1) Cell 1996 85 721 732 10.1016/S0092-8674(00)81238-6 8646780

[B41] Yan Y Frisén J Lee MH Massagué J Barbacid M Ablation of the CDK inhibitor p57Kip2 results in increased apoptosis and delayed differentiation during mouse development Genes Dev 1997 11 973 983 10.1101/gad.11.8.973 9136926

[B42] Coppé JP Rodier F Patil CK Freund A Desprez PY Campisi J Tumor suppressor and aging biomarker p16(INK4a) induces cellular senescence without the associated inflammatory secretory phenotype J Biol Chem 2011 286 36396 36403 10.1074/jbc.M111.257071 21880712 PMC3196093

[B43] Shlopov BV Lie WR Mainardi CL Cole AA Chubinskaya S Hasty KA Osteoarthritic lesions: involvement of three different collagenases Arthritis Rheum 1997 40 2065 2074 10.1002/art.1780401120 9365097

[B44] Imai K Dalal SS Hambor J Mitchell P Okada Y Horton WC D'Armiento J Bone growth retardation in mouse embryos expressing human collagenase 1 Am J Physiol Cell Physiol 2007 293 C1209 C1215 10.1152/ajpcell.00213.2007 17652426

[B45] Gack S Vallon R Schmidt J Grigoriadis A Tuckermann J Schenkel J Weiher H Wagner EF Angel P Expression of interstitial collagenase during skeletal development of the mouse is restricted to osteoblast-like cells and hypertrophic chondrocytes Cell Growth Differ 1995 6 759 767 7669731

[B46] Benanti JA Williams DK Robinson KL Ozer HL Galloway DA Induction of extracellular matrix-remodeling genes by the senescence-associated protein APA-1 Mol Cell Biol 2002 22 7385 7397 10.1128/MCB.22.21.7385-7397.2002 12370286 PMC135658

[B47] Macaluso M Montanari M Giordano A Rb family proteins as modulators of gene expression and new aspects regarding the interaction with chromatin remodeling enzymes Oncogene 2006 25 5263 5267 10.1038/sj.onc.1209680 16936746

[B48] Culley KL Hui W Barter MJ Davidson RK Swingler TE Destrument AP Scott JL Donell ST Fenwick S Rowan AD Young DA Clark IM Class I histone deacetylase inhibition modulates metalloproteinase expression and blocks cytokine-induced cartilage degradation Arthritis Rheum 2013 65 1822 1830 10.1002/art.37965 23575963

[B49] Mudhasani R Zhu Z Hutvagner G Eischen C Lyle S Hall L Lawrence J Imbalzano A Jones S Loss of miRNA biogenesis induces p19Arf-p53 signaling and senescence in primary cells J Cell Biol 2008 181 1055 1063 10.1083/jcb.200802105 18591425 PMC2442212

[B50] Kobayashi T Lu J Cobb BS Rodda SJ McMahon AP Schipani E Merkenschlager M Kronenberg HM Dicer-dependent pathways regulate chondrocyte proliferation and differentiation Proc Natl Acad Sci U S A 2008 105 1949 1954 10.1073/pnas.0707900105 18238902 PMC2538863

[B51] Lal A Kim HH Abdelmohsen K Kuwano Y Pullmann R Jr Srikantan S Subrahmanyam R Martindale JL Yang X Ahmed F Navarro F Dykxhoorn D Lieberman J Gorospe M p16(INK4a) translation suppressed by miR-24 PLoS One 2008 3 e1864 10.1371/journal.pone.0001864 18365017 PMC2274865

[B52] Sun F Wang J Pan Q Yu Y Zhang Y Wan Y Li X Hong A Characterization of function and regulation of miR-24-1 and miR-31 Biochem Biophys Res Commun 2009 380 660 665 10.1016/j.bbrc.2009.01.161 19285018

[B53] Brunner S Herndler-Brandstetter D Arnold CR Wiegers GJ Villunger A Hackl M Grillari J Moreno-Villanueva M Bürkle A Grubeck-Loebenstein B Upregulation of miR-24 is associated with a decreased DNA damage response upon etoposide treatment in highly differentiated CD8(+) T cells sensitizing them to apoptotic cell death Aging Cell 2012 11 579 587 10.1111/j.1474-9726.2012.00819.x 22435726 PMC3427896

[B54] Hassan MQ Gordon JA Beloti MM Croce CM van Wijnen AJ Stein JL Stein GS Lian JB A network connecting Runx2, SATB2, and the miR-23a 27a 24–2 cluster regulates the osteoblast differentiation program Proc Natl Acad Sci U S A 2010 107 19879 19884 10.1073/pnas.1007698107 20980664 PMC2993380

[B55] Pajcini KV Corbel SY Sage J Pomerantz JH Blau HM Transient inactivation of Rb and ARF yields regenerative cells from postmitotic mammalian muscle Cell Stem Cell 2010 7 198 213 10.1016/j.stem.2010.05.022 20682446 PMC2919350

[B56] van der Kraan PM van den Berg WB Chondrocyte hypertrophy and osteoarthritis: role in initiation and progression of cartilage degeneration? Osteoarthritis Cartilage 2012 20 223 232 10.1016/j.joca.2011.12.003 22178514

[B57] Braun H Schmidt BM Raiss M Baisantry A Mircea-Constantin D Wang S Gross ML Serrano M Schmitt R Melk A Cellular senescence limits regenerative capacity and allograft survival J Am Soc Nephrol 2012 23 1467 1473 10.1681/ASN.2011100967 22797186 PMC3431409

[B58] Obernosterer G Martinez J Alenius M Locked nucleic acid-based in situ detection of microRNAs in mouse tissue sections Nat Protoc 2007 2 1508 1514 10.1038/nprot.2007.153 17571058

