# Supplementary figures and images for: p16INK4a and its regulator miR-24 link senescence and chondrocyte terminal differentiation-associated matrix remodeling in osteoarthritis
Source: Arthritis Res Ther. 2014 Feb 27;16(1):R58. doi: 10.1186/ar4494 (PMC4060445; doi:10.1186/ar4494)

**A**

p16<sup>INK4a</sup> staining

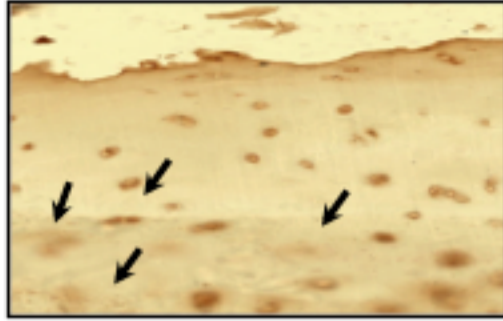

**B**

miR-24 staining

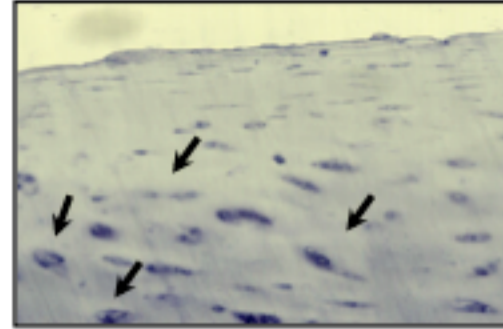

Superficial  
zone

Intermediate  
layer

Supplement: Additional file 1 — p16INK4a and miR-24 are reversely correlated in osteoarthritis (OA) articular cartilage. (A) p16INK4a immunohistochemistry (IHC) on a cryosection of OA cartilage comprising superficial and intermediate layer. (B) miR-24 in situ hybridization on an adjacent section of the same OA cartilage sample. Results are representative of experiments on two OA cartilage samples. Chondrocytes expressing either p16INK4a or miR-24 are marked by arrows showing mutual exclusion. OA cartilage samples were fixed with 4% paraformaldehyde during 3 hours at 4°C. After fixation, samples were placed in PBS with sucrose 30% during 24 hours at 4°C. The next day, PBS-sucrose 30% was replaced with Tek OCT solution and the samples were stored at −80°C. OA cartilage samples were sectioned at 13 μm and collected on Superfrost PLUS slides. In situ hybridization experiment was performed as described [58]. LNA DIG-hsa-miR-24 probe and DIG-has-miR-141c (as negative control) were purchased from Exiquon (Copenhagen, Denmark) and diluted at 1pM. Alkaline phosphatase conjugated anti-DIG- antibody was diluted at 1:2,000 in blocking solution. This file can be viewed with Acrobat Reader. [file ar4494-S1.pdf]
